# Supplementary material for: A broadly tunable synthesis of linear α-olefins
Source: Nat Commun. 2017 Oct 31;8:1226. doi: 10.1038/s41467-017-01507-2 (PMC5663737; doi:10.1038/s41467-017-01507-2)
Supplement: Supplementary file 1 — Supplementary Information [file 41467_2017_1507_MOESM1_ESM.pdf]

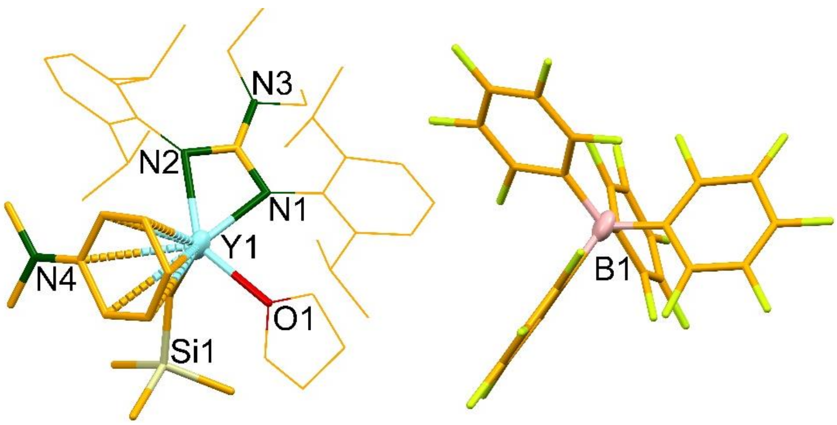

Supplementary Figure 1. Molecular structure of **1** with 50 % probability of thermal ellipsoids, hydrogen atoms are omitted for clarity. Selected bond lengths [Å] and angles [°]:Y1-O1 2.37(1), N1-Y1 2.27(1), N2-Y1 2.36(1), C30 Y1 2.33(2), N1-C13 1.34(2), N2-C13 1.36(2), C13-N3 1.40(2), "centroid: C34 C39 C35 C38 C36 C37" Y1 2.549; O1-Y1-C30 100.7(6), N1-Y1-N2 58.1(4), N1-C13-N2 113(1), O1-Y1-N1 86.3(5).

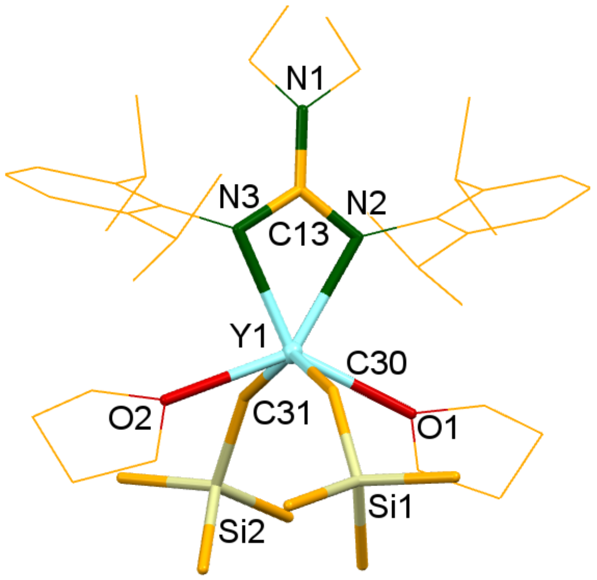

Supplementary Figure 2. Molecular structure of the precatalyst with 50% probability of thermal ellipsoids, hydrogen atoms are omitted for clarity. Selected bond lengths [Å] and angles [°]:O1-Y1 2.409(6), Y1-O2 2.448(6), C31-Y1 2.421(8), C30-Y1 2.439(9), N2-Y1 2.361(6), N3-Y1 2.347(6), N2-C13 1.35(1), N3-C13 1.34(1), N1-C13 1.38(1); O1-Y1-C31 85.5(2), C31-Y1-O2 80.6(2), O2-Y1-C30 84.8(2); C30-Y1-O1 82.6(2), N3-Y1-N2 55.9(2), N3-C13-N2 110.0(7)

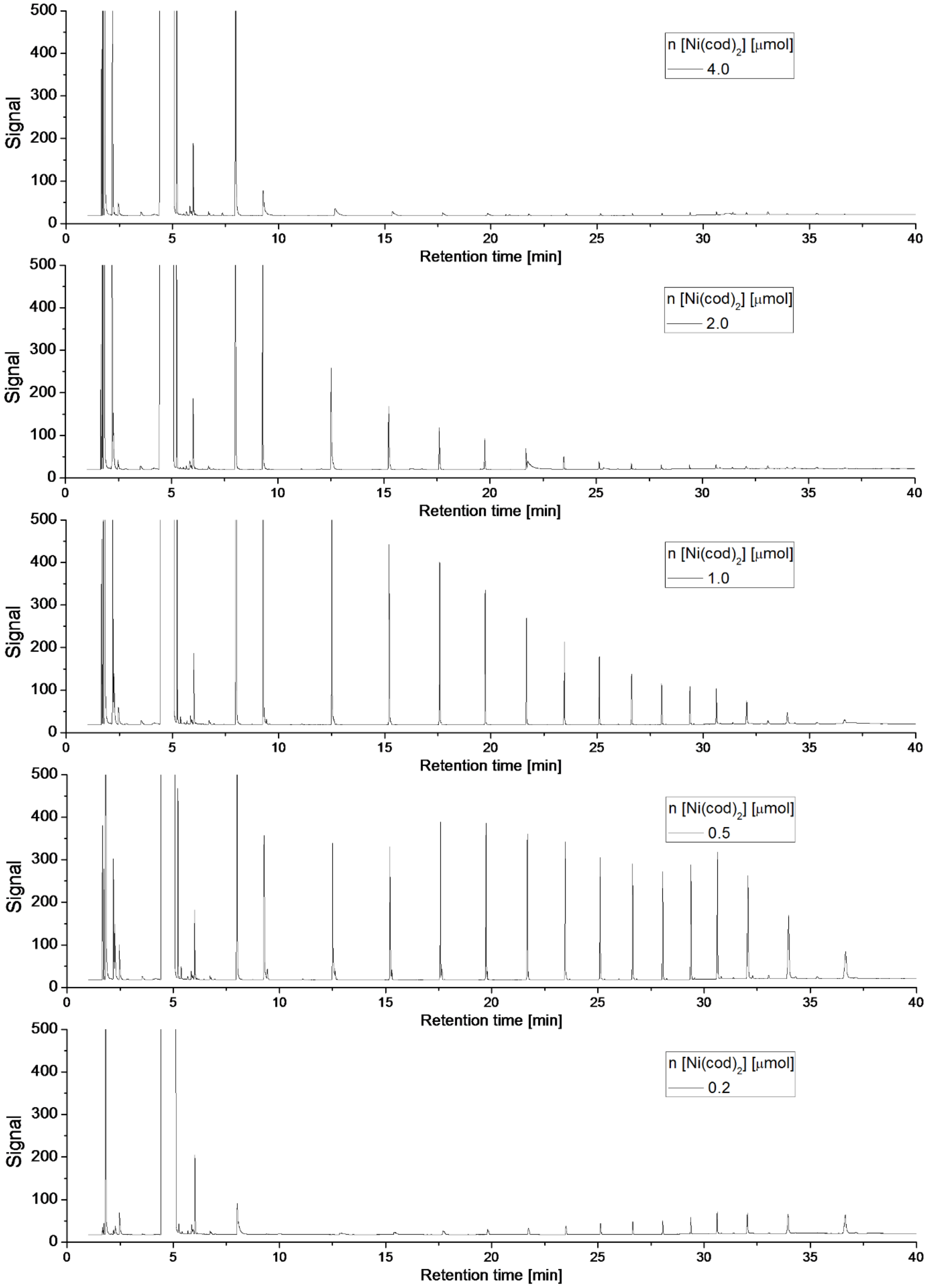

Supplementary Figure 3. Selected gas chromatograms of the products obtained from experiments described in Table 2, (entry 6-9).

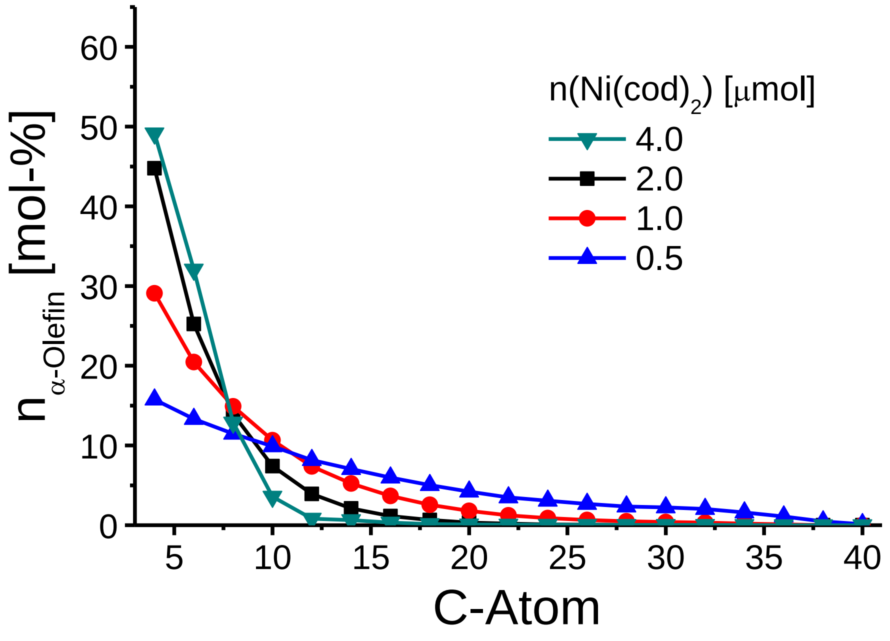

Supplementary Figure 4. Selected product distributions in mol-% obtained from experiments described in Table 2 (entry 3, 6-8).

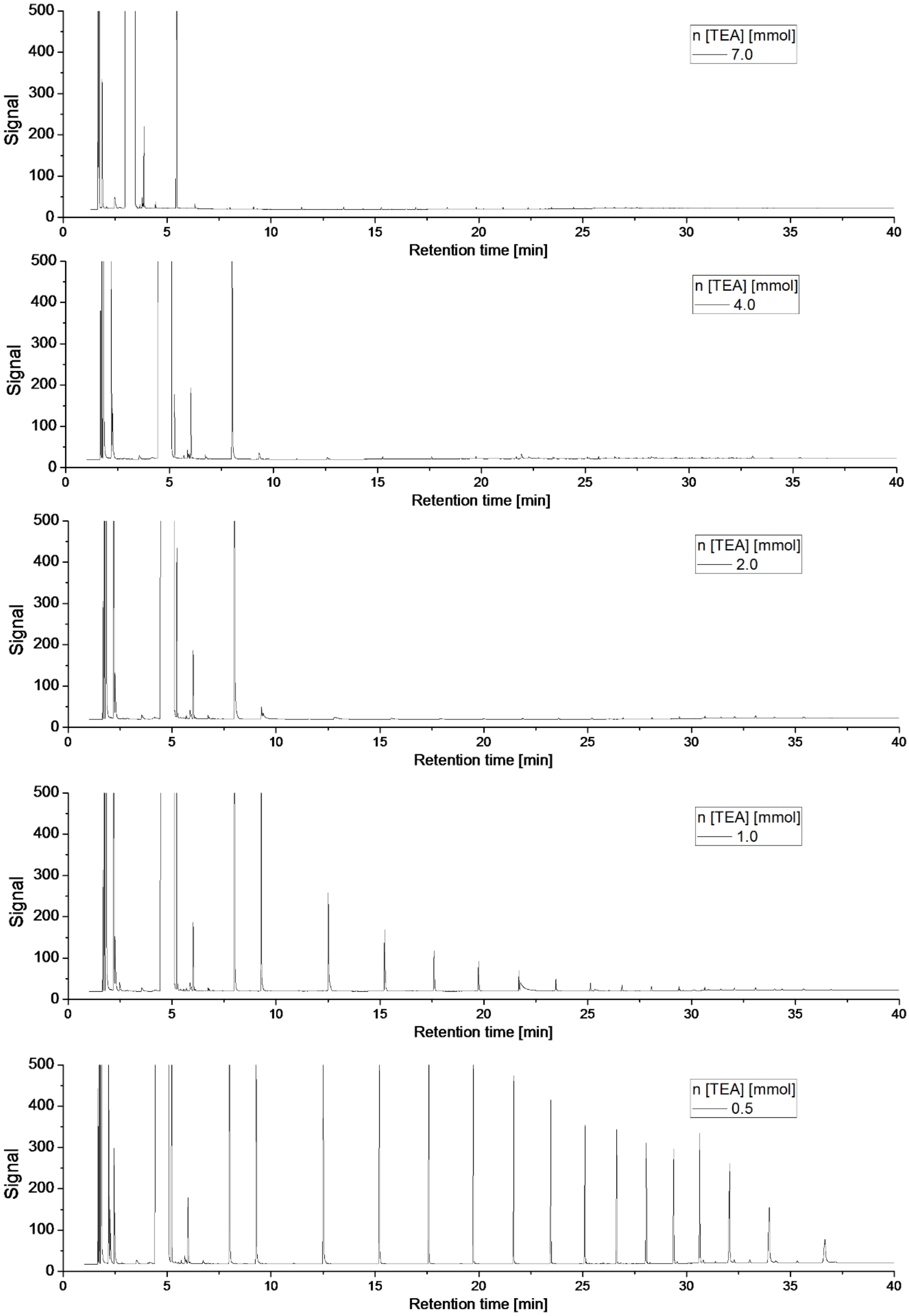

Supplementary Figure 5. Selected gas chromatograms of the products obtained from experiments described in Table 2, (entry 3, 10, 12, 13 and 14).

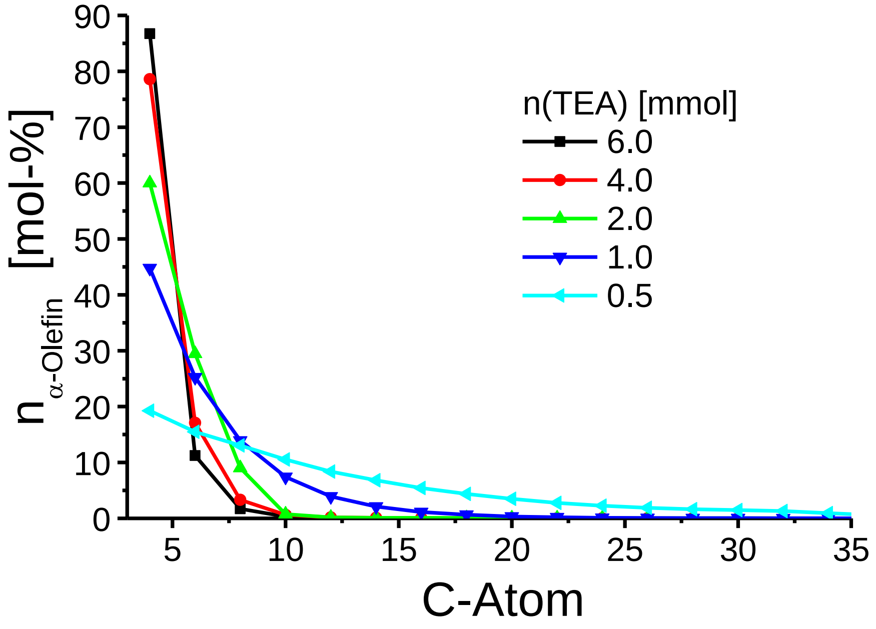

Supplementary Figure 6. Selected product distributions in mol-% obtained from experiments described in Table 2, (entry 10-14).

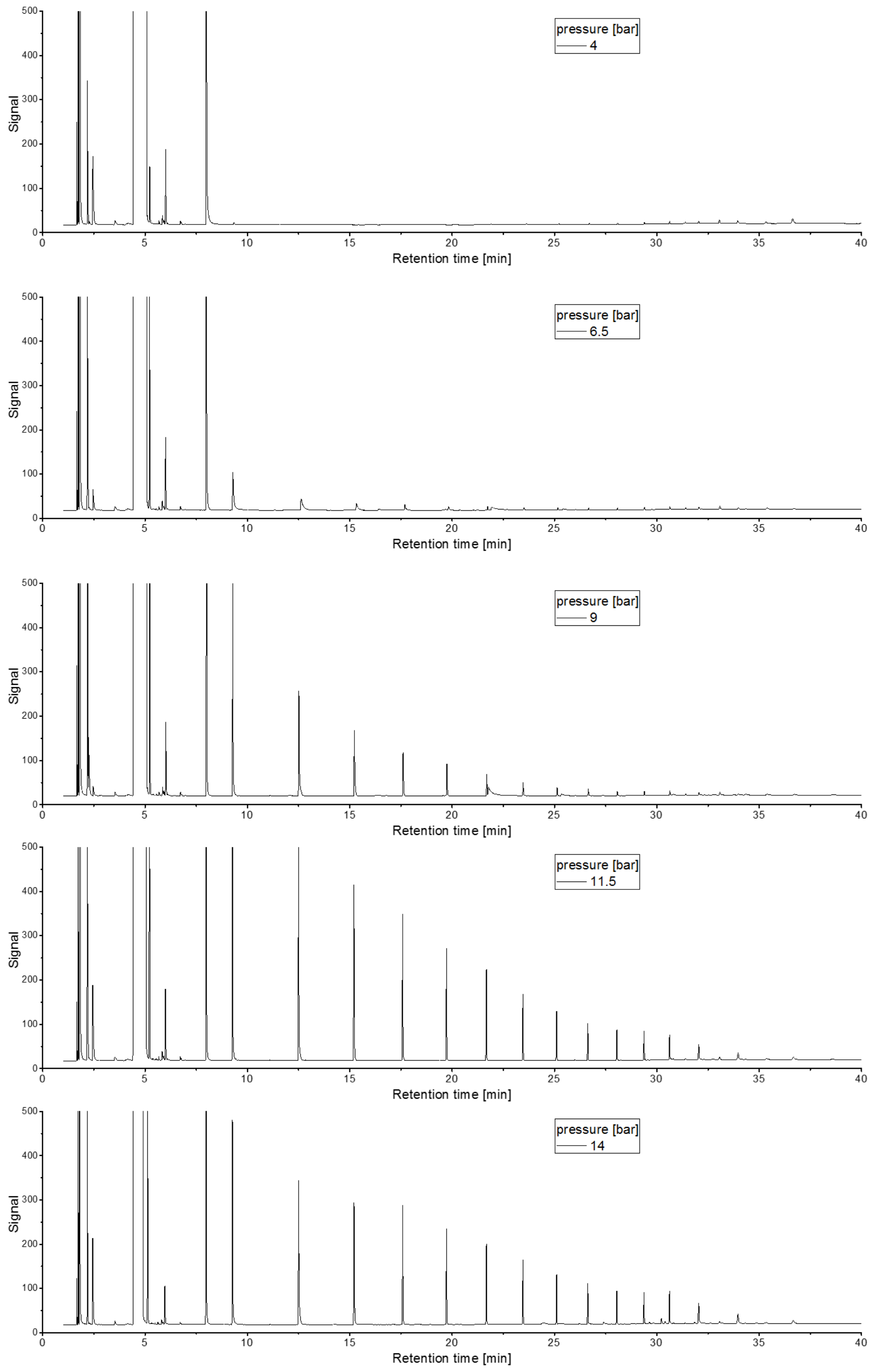

Supplementary Figure 7. Selected gas chromatograms of the products obtained from experiment described in Table 2, (entry 3, 15-18).

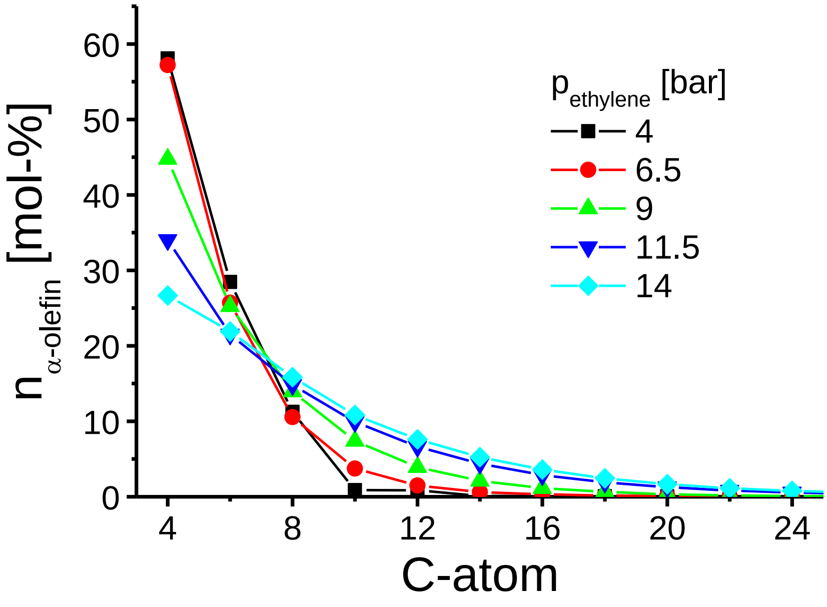

Supplementary Figure 8. Selected product distribution in mol-% obtained from experiments described in Table 2, (entry 3, 15-18).

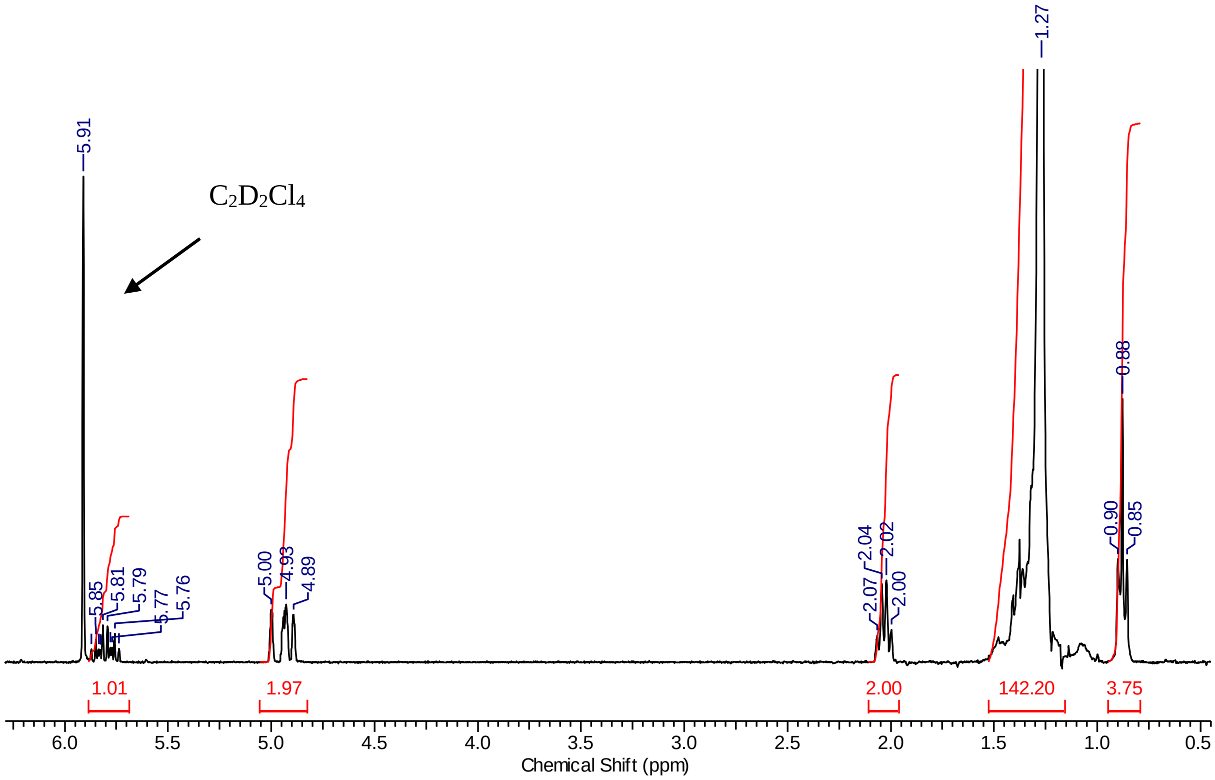

Supplementary Figure 9. ^{1}H NMR spectrum (393 K, $C_2D_2Cl_4$, 300 MHz) obtained from the extreme run with Y/Al/Ni (1/100/0.02) described in Figure 1d, Table 2, (entry 9).

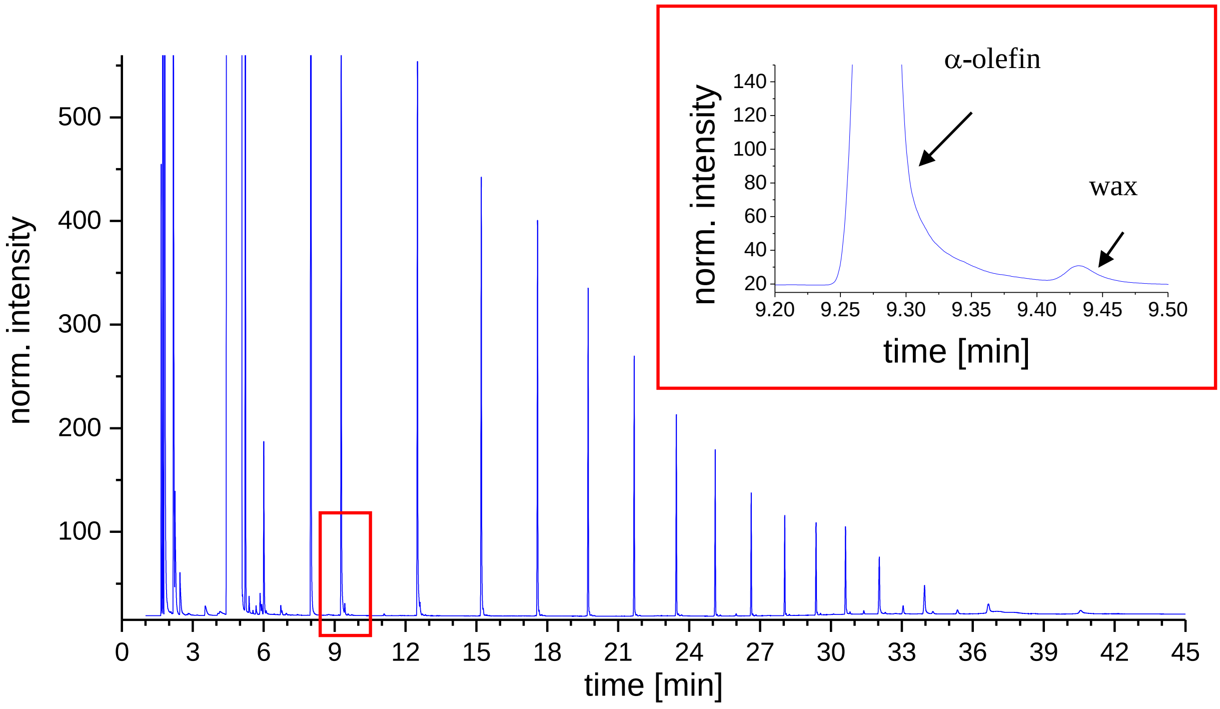

Supplementary Figure 10. A typical gas chromatogram obtained from experiment entry 3 in Table 2. Schulz Flory equation[1] used for fitting calculations (Solver Add-in in Microsoft Excel was used for a least-square fit of the obtained molar distributions): $C_{\mathrm{p}} = \mathrm{c} * (1-\alpha) * \alpha^{(n-1)}$, α = probability of chain propagation, c = total amount (moles) of produced α-olefin, C_{p} = amount (moles) of α-olefin with n monomer units inserted (n = 1 means the formation of 1-butene, due to an ethyl group is the starting chain length).

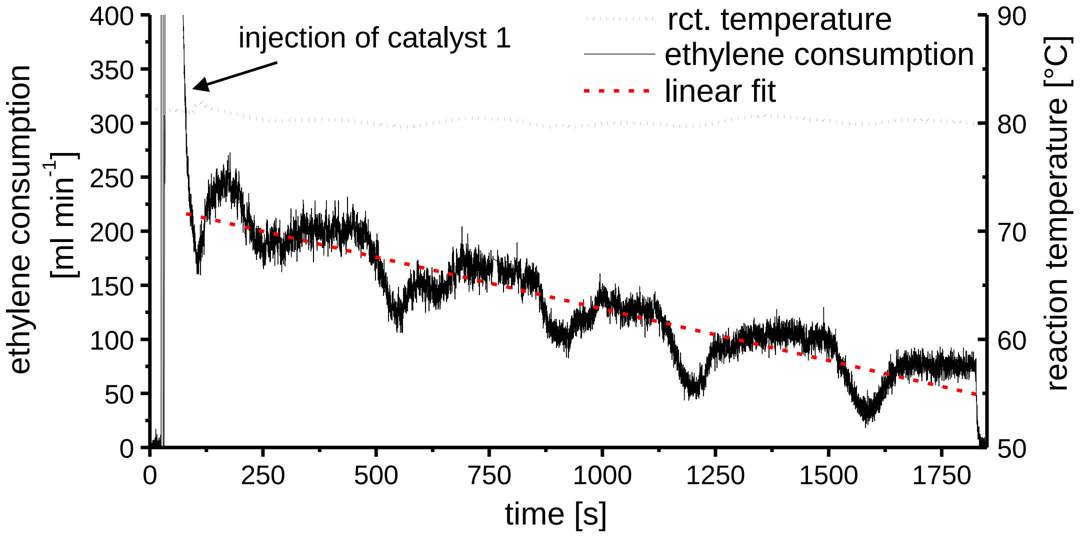

Supplementary Figure 11. Ethylene consumption [ml min^{-1}] (black line, left y-axis) over time and reaction temperature (dotted blue line, right y-axis) [°C] of the CCTP run with catalyst 1, TEA and $Ni(cod)_2$ described in Table 1, Entry 9. Linear fit of the ethylene consumption: y = -0.0956 (± 3.272 10^{-4}) ml min^{-1} s^{-1} * x + 223.8 (± 0.3534) ml min^{-1}. The half life (time after which 50% of the initial ethylene consumption is reached) of the system is approx. 19 minutes.

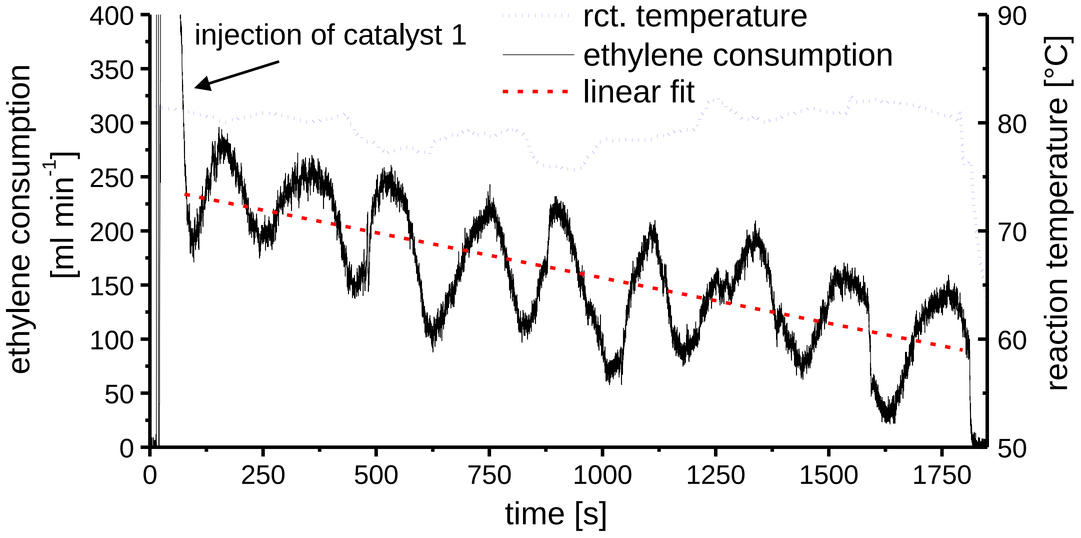

Supplementary Figure 12. Ethylene consumption [ml min^{-1}] (black line, left y-axis) over time of the CCTP run with catalyst 1 and TEA described in Table 1, Entry 6. Linear fit of the ethylene consumption: y = -0.083 (± 6.146 10^{-4}) ml min^{-1} s^{-1} * x + 240.3 (± 0.651) ml min^{-1}. The half life (time after which 50% of the initial ethylene consumption is reached) of the system is approx. 24 minutes

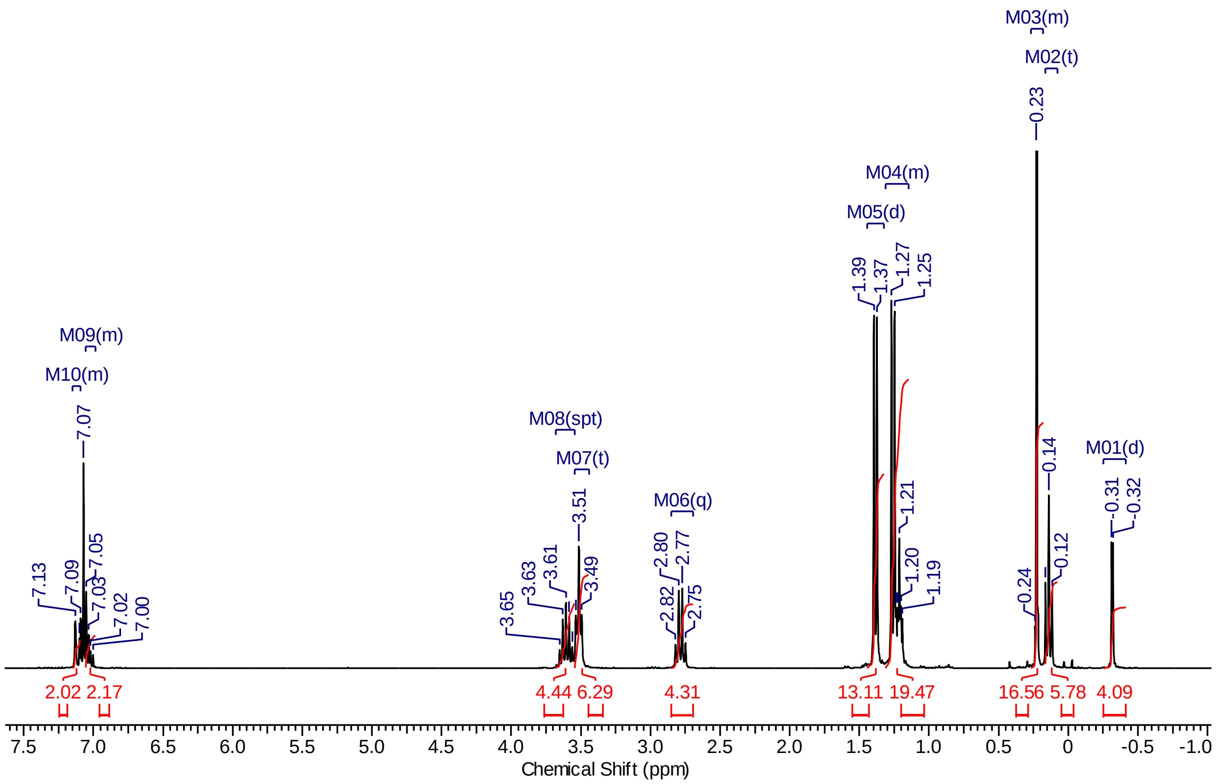

Supplementary Figure 13. ^{1}H NMR spectrum (298 K, C_6D_6, 300 MHz) of the Yttrium based precatalyst.

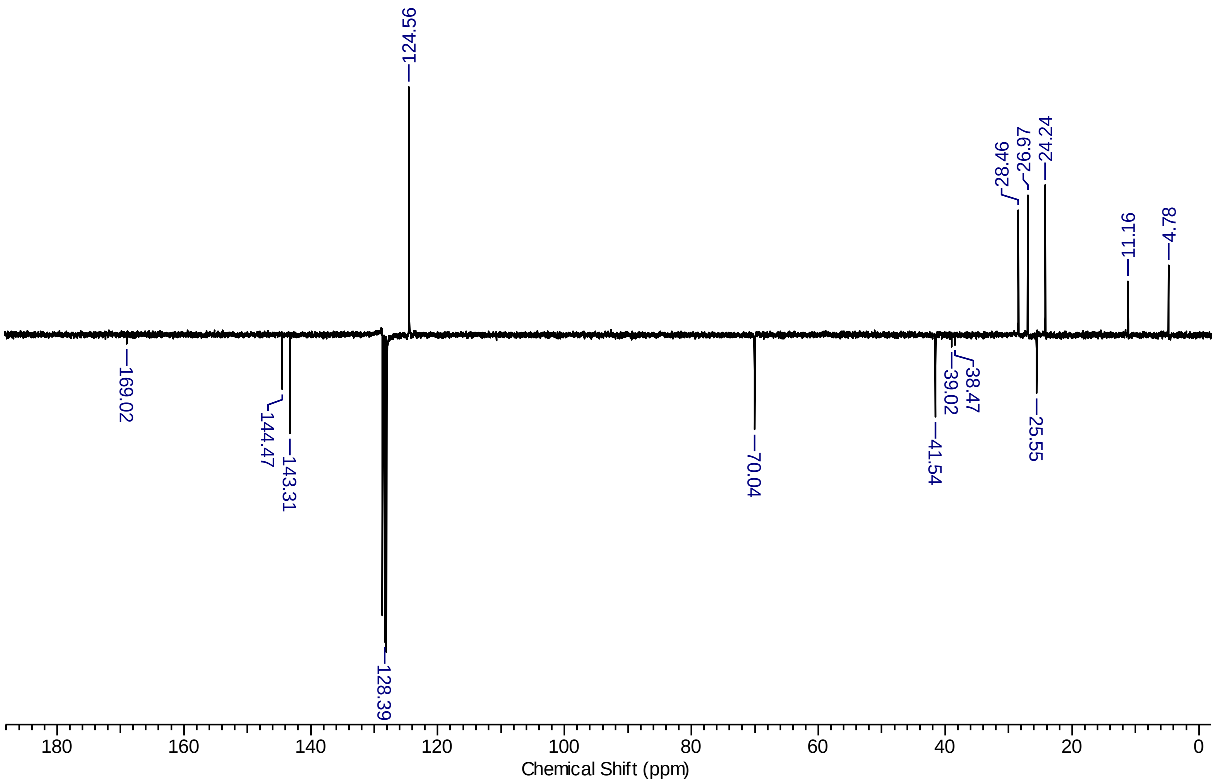

Supplementary Figure 14 . ^{13}C NMR APT spectrum (75 MHz, 298 K, C_6D_6) of the Yttrium based precatalyst.

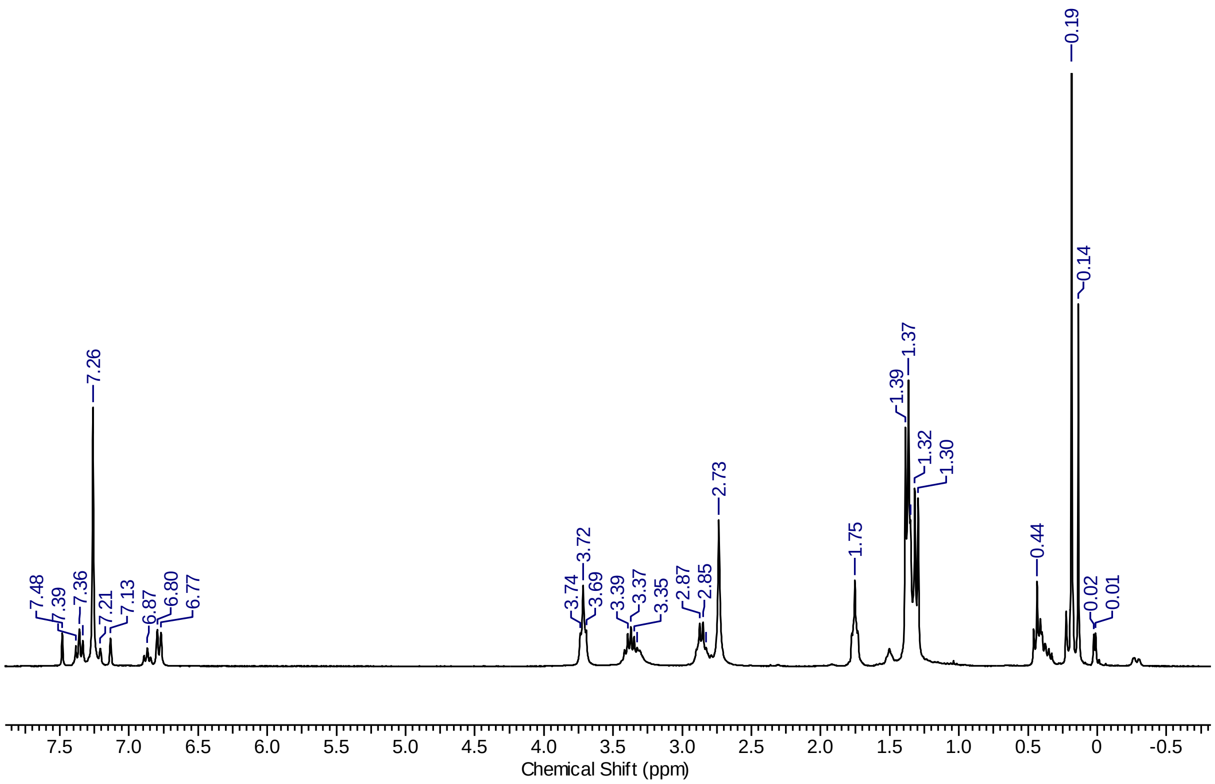

Supplementary Figure 15. ^{1}H NMR spectrum (298 K, C_6D_5Br, 300 MHz) of the reaction of the catalyst **1**.

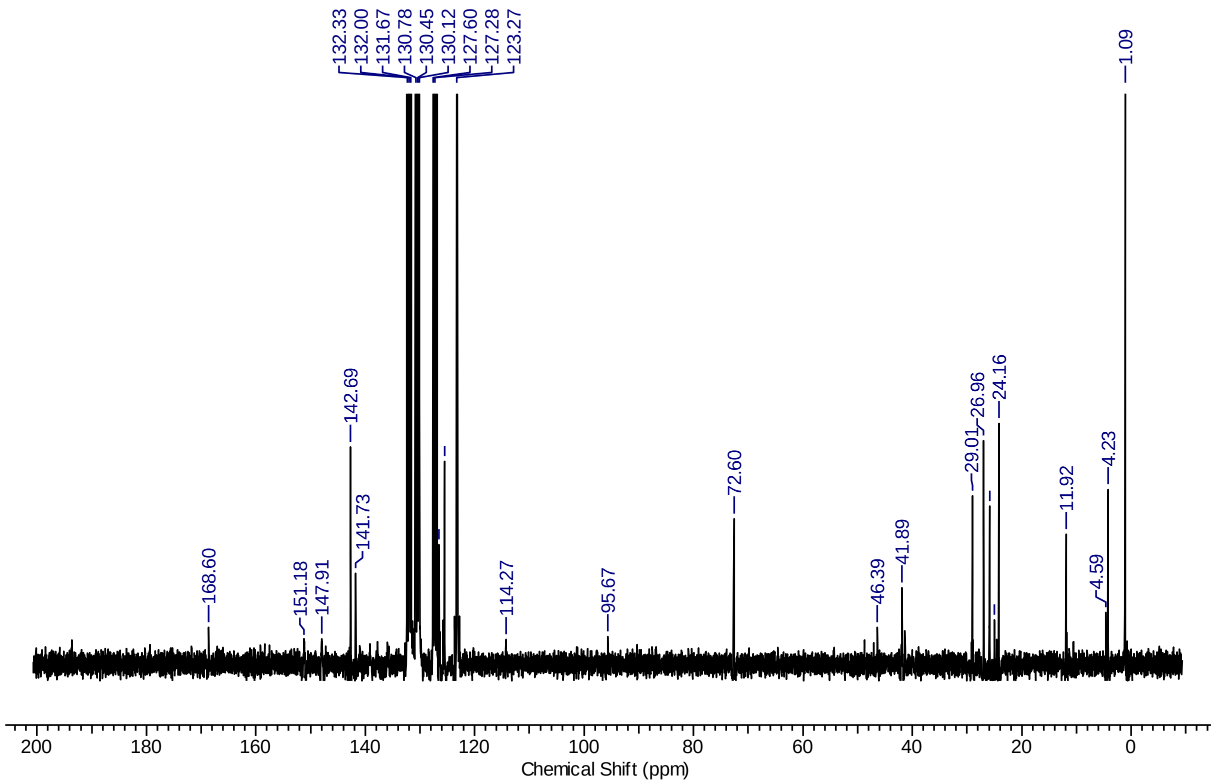

Supplementary Figure 16. ^{13}C NMR spectrum (298K, C_6D_5Br, 75 MHz) of the catalyst **1** in bromobenzene-d5.

Supplementary Table 1. CCTP runs using the catalyst **1** with TEA

Entry	$c(AlEt_3)$ [mmol L^{-1}]	Al/Y	V_{eth} [L_n]	productivity[a]	M_n [g mol^{-1}]	M_w/M_n
1	16	400	1.4	720	120	1.1
2	8	200	1.9	950	310	1.1
3	4	100	3.1	1600	2160	1.1
4	2	50	5.2	2800	4700	1.2

Reaction conditions: catalyst n = 10 μmol, p_{eth} = 9 bar, T = 80 °C, t = 1800s, $V_{toluene}$ = 250 mL; [a] [$kg_{ehtylene}$ mol^{-1} h^{-1}]

Supplementary Methods

General Methods

All manipulations of air sensitive compounds were performed with exclusion of oxygen and moisture using standard Schlenk techniques or a nitrogen or argon filled glove box (mBraun) with a high capacity circulator (< 0.1 ppm O_2). Deuterated solvents were received from Cambridge Isotope Laboratories. All solvents used for air and moisture sensitive reactions were dried and purified by distillation from Na/benzophenone or CaH_2 (halogenated solvents) under argon atmosphere.

GC, GC-MS, GPC and NMR analysis

The toluene soluble fractions were analyzed with an Agilent 6890N gas chromatograph (GC), equipped with a FID and a 30 m HP-5 column, and with an Agilent 7890A gas chromatograph (GC) combined with an Agilent 5975C mass selective detector (MSD), equipped with a TCD, a 30 m HP-5 column and a Triple-Axis-Detector, using cumene as internal standard.

Gel permeation chromatography (GPC) analyses were carried out on an Agilent (Polymer Laboratories Ltd.) PL-GPC 220 high temperature chromatographic unit equipped with DP and RI detectors and three linear mixed bed columns (PSS POLEFIN Linear XL). GPC analysis was performed at 150 °C using 1,2,4-trichlorobenzene as the mobile phase. The samples were prepared by dissolving the polymer (0.1 wt-%) in the mobile phase solvent in an external oven and the solutions were run without filtration. The molecular weights of the samples were referenced to polystyrene standards (Mw = 518-2,500,000 g mol^{-1}, K = 12.100 and Alpha = 0.707) and were corrected with K and Alpha values for linear PE (K = 40.600 and Alpha = 0.725).

All ^{1}H and ^{13}C spectra were recorded with Varian INOVA 300 (^{1}H: 300 MHz, ^{13}C: 75.4 MHz) spectrometer. The ^{1}H and ^{13}C NMR spectra were referenced internally by using the residual solvent resonances. Chemical shifts (δ) are reported in ppm.

X-ray Crystallography

X-Ray crystal structure analyses were performed with a STOE IPDS-II and a STOE STADIVARI diffractometer [λ(Mo-Kα)= 0.71073 Å] equipped with an Oxford Cryostream low temperature unit. Structure solution and refinement were accomplished with SIR97[2], SHELXL-97[3,4] and WinGX[5,6]

Elemental analysis

Elemental Analyses (C, H, N): Elemental analyses were carried out with an Elementar Vario EL III apparatus.

Polymerization

Toluene (Aldrich, anhydrous, 99.8%) and ethylene (Linde AG polymer grade) used for ethylene polymerization was passed over columns of BASF R3–11-supported Cu oxygen scavenger and Al_2O_3 (Fischer Scientific).

Triethyl aluminum ($AlEt_3$, TEA, SASOL Germany GmbH), $[Ni(cod)_2]$ (Sigma Aldrich), $[Ni(acac)_2]$ (ABCR), $[Ni(CO_2(CH_2)_{16}CH_3)_2]$ (ABCR), Dimethylanilinium tetrakis (pentafluorophenyl)borate (ABCR) were used without any further purification.

The oligomerization runs were performed in an 1 L stainless steel autoclave (Büchiglasuster) equipped with a mechanical stirrer (at 1000 rpm). It is equipped with a temperature controllable jacket, combining electrical heating and water cooling. Ethylene flow was monitored with a Bronkhorst High-Tech EI-Flow. All catalysts and CTA compounds were applied as stock solutions in toluene.

Synthesis and characterization of the metal complexes

The preparation of the precatalyst was carried out as described in literature.[7,8] The metal precursor $Y(CH_2SiMe_3)_3 * 2$ THF[9] and the 2,3-bis(2,6-diisopropylphenyl)-1,1-diethylguanidine ligand[101112] were synthesized according to published literature.

Synthesis and characterization of the Yttrium based precatalyst

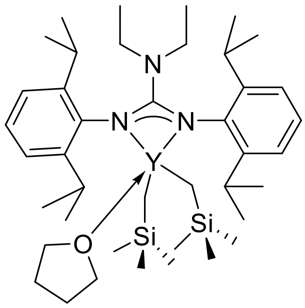

This complex can be obtained with either one or two THF molecules coordinating the Y center[13]: To a solution of $Y(CH_2SiMe_3)_3 * 2$ THF (2.42 g, 4.90 mmol) in hexane (75 mL) 2,3-bis(2,6-diisopropylphenyl)-1,1-diethylguanidine (2.13 g, 4.90 mmol) was added at rt. and stirred overnight (18 h). After a subsequent filtration and removal of the solvent under reduced pressure to approx. 20 mL, colourless crystals suitable for x-ray were obtained after 2 days at -32 °C. Isolated yield: 3.11 g, 4.04 mmol, 82 %. Elem. Anal. calcd. for $C_{41}H_{77}N_3OSi_2Y$ (770.14 $gmol^{-1}$): C, 63.94; H, 9.69; N, 5.46; found: C, 63.40; H, 9.53; N, 5.44.

^1H NMR (300 MHz, C_6D_6, 298 K) δ ppm -0.32 (d, *J*=3.51 Hz, 4 H) 0.14 (t, *J*=7.03 Hz, 6 H) 0.18 - 0.27 (bs, 18 H) 1.14 - 1.31 (m, 16 H) 1.38 (d, *J*=6.44 Hz, 12 H) 2.78 (q, *J*=7.60 Hz, 4 H) 3.51 (m, *J*=6.44 Hz, 4 H) 3.61 (spt, *J*=6.44 Hz, 4 H) 6.99 - 7.06 (m, 3 H) 7.09 - 7.15 (m, 3 H)

^{13}C NMR (75 MHz, C_6D_6, 298K): δ = 4.78 (6 C), 11.16(2 C), 24.24 (2 C), 25.55 (4 C), 26.97 (2C), 28.97 (2C), 38.47, 39.02 (2 C), 41.54 (2 C), 70.04 (2 C), 1124.56 (4 C), 143.31 (4 C,), 144.47 (2 C), 169.28 (1 C) ppm.

Synthesis and characterization of 1

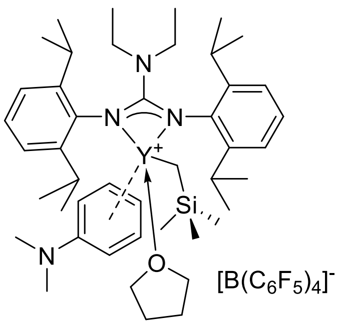

To a solution of the precatalyst (50.0 mg, 0.065 mmol) in 0.5 mL bromobenzene N,N-dimethylaniliniumtetrakis (pentafluorophenyl)borate (52.2 mg, 0.065 mmol) was added, mixed and cooled at -32 °C for 5 days. After addition of a 0.5 mL hexane layered above, suitable crystals for x-ray could be obtained. Isolated yield: 65 mg, 0.043 mmol, 67 %. Anal. calcd. for $C_{69}H_{74}BF_{20}N_4OSiY$ (1483.15 gmol^{-1}): C, 55.88; H, 5.03, N, 3.78; found: C, 55.98; H, 4.56; N, 3.71.

^{1}H NMR (300 MHz, C_6D_6, 298K): δ = 0.02 (d, *J*=3.51 Hz, 2 H), 0.16 (s, 9 H), 0.29 - 0.50 (m, 6 H), 1.27 - 1.43 (m, 24 H), 1.67 - 1.81 (m, 4 H), 2.73 (s, 6 H), 2.86 (q, *J*=7.03 Hz, 4 H), 3.19 - 3.45 (m, 4 H), 3.63 - 3.77 (m, 4 H), 6.78 (d, *J*=8.20 Hz, 2 H), 6.86 (m, 1 H), 7.19 – 7.41 (m, 9H).

^{13}C NMR (75 MHz, C_6D_6, 298K): δ = 1.09 (3 C), 4.23 (1C), 4.59 (1 C), 11.75 (1 C), 11.92 (2 C), 24.16 (2 C), 25.01 (1 C), 25.89 (2 C), 26.96 (2 C), 29.01 (2C), 41.38 (1 C), 41.89 (2C), 46.39 (1 C), 72.60 (2 C), 95.67 (1 C), 125.51 (2 C), 125.78 (1 C), 126.36 (1 C), 126.54 (2C), 141.73 (1 C), 142.69 (2 C), 147.91 (1 C), 151.24 (1C), 168.60 (1C).

Supplementary References

[1] Wesslau, H. Das Problem der Kettenlängenverteilung bei der stufenweisen metallorganischen Synthese. *Liebigs Ann. Chem.* **629,** 198–206 (1960).

[2] Altomare, A. *et al.* SIR 97. A new tool for crystal structure determination and refinement. *J. Appl. Crystallogr.* **32,** 115–119 (1999).

[3] Sheldrick, G.M. Crystal structure refinement with SHELXL. *Acta Crystallogr. Sec. C, Struct. Chem.* **71,** 3–8 (2015).

[4] Sheldrick, G.M. A short history of SHELX. *Acta Crystallogr., A, Found. Crystallogr.* **64,** 112–122 (2008).

[5] Farrugia, L.J. WinGX and ORTEP for Windows. An update. *J Appl Crystallogr* **45,** 849–854 (2012).

[6] Farrugia, L.J. WinGX suite for small-molecule single-crystal crystallography. *J. Appl. Crystallogr.* **32,** 837–838 (1999).

[7] Kretschmer, W.P. *et al.* Reversible chain transfer between organoyttrium cations and aluminum: synthesis of aluminum-terminated polyethylene with extremely narrow molecular-weight distribution. *Chem. Eur. J.* **12,** 8969–8978 (2006).

[8] Kretschmer, W.P., Bauer, T., Hessen, B. & Kempe, R. An efficient yttrium catalysed version of the "Aufbaureaktion" for the synthesis of terminal functionalised polyethylene. *Dalton Trans.* **39,** 6847–6852 (2010).

[9] Lappert, M.F. & Pearce, R. Stable silylmethyl and neopentyl complexes of scandium(III) and yttrium(III). *J. Chem. Soc., Chem. Commun.,* **4,** 126 (1973).

[10] Jin, G. *et al.* Synthesis and characterisation of bulky guanidines and phosphaguanidines. Precursors for low oxidation state metallacycles. *New J. Chem.* **33,** 64–75 (2009).

[11] Scheer, M. The coordination chemistry of group 15 element ligand complexes--a developing area. *Dalton Trans.,* **33,** 4372–4386 (2008).

[12] Zhou, M., Zhang, S., Tong, H., Sun, W.-H. & Liu, D. Synthesis, structure and catalytic properties of a novel zirconium guanidinato complex [Zr{ArNC(NMe_2)N($SiMe_3$)}(μ^2-Cl)Cl_2]$_2$[Ar=2,6-iPr_2-C_6H_3]. *Inorg. Chem. Commun.* **10**, 1262–1264 (2007).

[13] Bambirra, S., van Leusen, D., Meetsma, A., Hessen, B. & Teuben, J.H. Yttrium alkyl complexes with a sterically demanding benzamidinate ligand. Synthesis, structure and catalytic ethene. *Chem. Commun.,* **4,** 522–523 (2003)
